# The BrEasT cancer afTER-CARE (BETTER-CARE) programme to improve breast cancer follow-up: design and feasibility study results of a cluster-randomised complex intervention trial

**DOI:** 10.1186/s13063-024-08614-8

**Published:** 2024-11-14

**Authors:** Anna Schäfer, Julia Wendel, Isabella Franke, Armin Bauer, Harald Baumeister, Eileen Bendig, Sara Y. Brucker, Thomas M. Deutsch, Patricia Garatva, Kirsten Haas, Lorenz Heil, Klemens Hügen, Helena Manger, Rüdiger Pryss, Viktoria Rücker, Jessica Salmen, Andrea Szczesny, Carsten Vogel, Markus Wallwiener, Achim Wöckel, Peter U. Heuschmann, the BETTER-CARE Study Group, Alexander Ast, Kerstin Belke, Björn Beurer, Petra Bolkenius, Kristina Freese, Nora Frumkin, Karsten Gnauert, Heiko Graf, John Hackmann, Corinna Hartmann, Tobias Hesse, Melanie Hopp, Elke Keil, Antje Lehnert, Cordula Müller, Christoph Mundhenke, Hue Phan Niestroj, Antje Nixdorf, Kilian Pankert, Sibylle Perez, Julia Radosa, Daniela Rezek, Jens-Paul Seldte, Gabriele Stalzer, Zuzana Sykorova, Martin Tenger, Léa Volmer, Katharina Würfel, Andreas Zorr

**Affiliations:** 1 Institute of Clinical Epidemiology and Biometry, Julius-Maximilians-Universität Würzburg, Würzburg, Germany; 2 Department of Gynecology and Obstetrics, University Hospital Würzburg, Würzburg, Germany; 3 Institute Women’s Health GmbH, Tübingen, Germany; 4 Department of Clinical Psychology and Psychotherapy, Institute of Psychology and Education, University of Ulm, Ulm, Germany; 5 Department of Women’s Health, University Women’s Hospital Tübingen, Tübingen, Germany; 6 University Hospital Heidelberg, Heidelberg, Germany; 7 University Hospital Würzburg, Clinical Trial Center Würzburg, Würzburg, Germany; 8 Faculty of Business Management and Economics, Julius-Maximilians-Universität Würzburg, Würzburg, Germany; 9 Institute for Medical Data Science, University Hospital Würzburg, Würzburg, Germany; 10 University Women’s Hospital Halle, Halle, Germany

**Keywords:** Breast cancer, Complex intervention, Follow-up care, Study protocol, Pilot study

## Abstract

**Background:**

The risk of breast cancer patients for long-term side effects of therapy such as neurotoxicity and cardiotoxicity as well as late effects regarding comorbidities varies from individual to individual. Personalised follow-up care concepts that are tailored to individual needs and the risk of recurrences, side effects and late effects are lacking in routine care in Germany.

**Methods:**

We describe the methodology of BETTER-CARE, a parallel-arm cluster-randomised controlled trial conducted at 15 intervention and 15 control centres, aiming to recruit 1140 patients, and the results of the pilot phase. The needs- and risk-adapted complex intervention, based on existing development frameworks, includes a multidisciplinary network and digital platforms for symptom and need documentation and just-in-time adaptive interventions. The control group comprises usual care according to clinical guidelines. The primary outcome is health-related quality of life (EORTC QLQ-C30 global health), and secondary outcomes include treatment adherence.

**Results:**

The 2-month pilot phase comprising 16 patients in one intervention and one control pilot centre demonstrated the feasibility of the BETTER-CARE approach.

**Discussion:**

BETTER-CARE is a feasible intervention and study concept, investigating individualised needs- and risk-adapted breast cancer follow-up care in Germany. If successful, the approach could be implemented in German routine care.

**Trial registration:**

German Clinical Trial Register DRKS00028840. Registered on April 2022.

**Supplementary Information:**

The online version contains supplementary material available at 10.1186/s13063-024-08614-8.

## Introduction

Breast cancer is the most common malignant tumour in Germany and worldwide [1, 2]. Mortality rates for breast cancer are falling and breast cancer survivors report frequently unmet needs for long-term support [3–5]. In addition to somatic side effects of the therapy, such as neurotoxicity and cardiotoxicity, lymphoedema or pain [6–8], increased tumour-related stress or mental illnesses such as anxiety and depression play a central role in follow-up care. These factors can have negative consequences for the disease course, including an increase in the frequency and duration of hospital stays, lower adherence to treatment recommendations, difficulties in coping with the disease, reduced quality of life and an increase in mortality [9–14]. So far, an individualised breast cancer follow-up care concept is lacking in Germany, although required by current clinical guidelines [15].

Within the framework of the German guideline of diagnosis and care of breast cancer (“AWMF S3 Leitlinie”) and the development of Cancer Survivorship Programmes, a needs- and risk-adapted follow-up care is recommended after completion of primary treatment [15–17]. Internationally, the concept of the Breast Care Nurse is increasingly being used to identify patients' needs and to support the delivery of appropriate care [18]. In Germany, acute breast cancer treatment is usually performed in Breast Cancer Centres, whereas follow-up care is delivered by gynaecologists in outpatient care. The exchange between specialists in outpatient care and Breast Cancer Centres is made difficult as different patient record files are used which are not automatically exchanged [19]. Further, fixed time intervals for follow-up care independent of patients` risks are currently standard of German follow-up care. Limited healthcare capacities must be balanced between patients who suffer from reduced quality of life due to high fear of recurrence and patients who have received aggressive therapies due to the high risk of recurrence and suffer from side and late effects [15, 20]. It is therefore particularly important to address patients' quality of life in addition to survival to take into account existing late effects of therapy and unmet needs [21]. Internet-based interventions can be an effective way to support people with cancer. They can be used to prevent mental disorders in general, to support lifestyle changes and medical treatment in people with chronic somatic diseases, or to support quality of life and stress reduction in cancer patients, but more evidence for breast cancer survivors is needed [22–24].

To date, data on the effect of a multidisciplinary care network supplemented by digital applications to provide needs- and risk-based individualised follow-up care on improving the quality of life in patients with breast cancer is lacking. Here, we present the study protocol and the results of the feasibility study of the BrEasT cancer afTER CARE follow-up and programme (BETTER-CARE), currently being tested as a complex intervention study in Germany to improve quality of life; reduce therapy-related toxicities, psychological distress and rehospitalization; improve progression-free survival; increase patient satisfaction; enhance participation in social and working life; and enhance compliance with appropriate adjuvant therapies.

## Methods

The BETTER-CARE study protocol was developed following the SPIRIT 2013 statement (see Additional file 1) [25]. BETTER-CARE is a parallel-arm cluster-randomised controlled trial with 30 clusters. The study is running from March 2023 until June 2025. A cluster is defined as Breast Cancer Centres, where acute care is provided for a defined source population. All German Breast Cancer Centres that were certified at enrolment by the German Cancer Society (“Deutsche Krebsgesellschaft”) and the German Society for Senology and that could export routine data via the Onkozert© software, as this was part of the study, were eligible and invited to the study by the German Cancer Society [26]. Breast Cancer Centres in Germany are assessed by the Certification Commission based on the following criteria, among others, for certification: Tumour conference with participation of defined disciplines and demonstration of imaging material, implementation of designated clinical guidelines and assurance of availability, number of primary breast cancer cases per year with initial certification ≥ 100 primary cases, 24-h accessibility of the main clinical cooperation partners, cooperation with psycho-oncology, social services, self-help, genetic counselling, gene analyses, physiotherapy, laboratory, palliative medicine [27]. In 2020, 284 sites were certified and a total of 61,356 primary breast cancer cases were treated [28]. Of the Breast Cancer Centres that expressed interest, a predetermined number of 30 Breast Cancer Centres were selected whose catchment areas did not overlap to avoid contamination bias. Furthermore, no other follow-up programme with digital applications should be offered regularly at the Breast Cancer Centre. Both university and non-university clinics were included. The size of the city and the centre were recorded, as these are potential confounding variables regarding the quality of care. Since the Breast Cancer Centres of the intervention group provide consulting and education, no blinding of patients and physicians is possible. In the control condition, patients receive usual care according to German guidelines and a study-specific assessment of primary and secondary outcomes. The study was registered on the German Clinical Trials Register (DRKS00028840) in April 2022.

### Participants

**Fig. 1:**
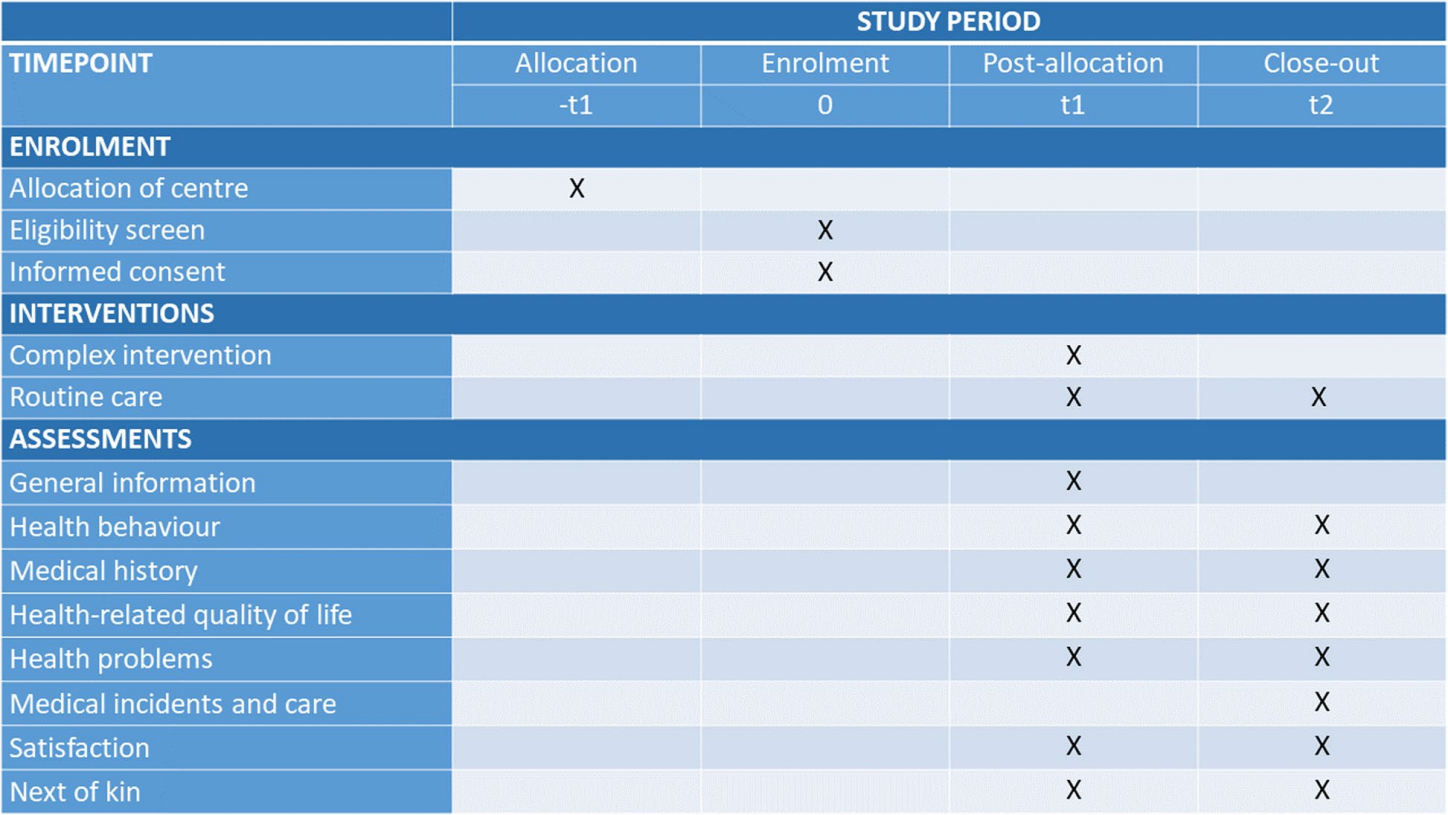
Schedule of enrolment, interventions, and assessments of the BETTER-CARE trial

The Breast Cancer Centres screen and enrol their patients into the allocated study programme (Fig. 1). Inclusion criteria are defined as follows: Breast cancer patients; age 18 years or older; no restriction to specific gender; end of curative primary treatment (defined as at the latest 10 weeks after completion of surgery and radiotherapy or after completion of (neo-) adjuvant chemotherapy, surgery and radiotherapy before starting or during the first 2 weeks of postneoadjuvant chemo/antibody therapy or (extended, combined) endocrine therapy); and consent to participation. Exclusion criteria comprise the diagnosis of metastatic breast carcinoma (palliative therapy approach) and a lack of digital infrastructure of the patient (defined as no access to a mobile device with Internet connection). Patients are additionally excluded when participating in another structured follow-up programme with an intervention via digital platforms (analogous to electronic health record or digital interventions like those used in the BETTER-CARE programme).

### Intervention programme

**Fig. 2:**
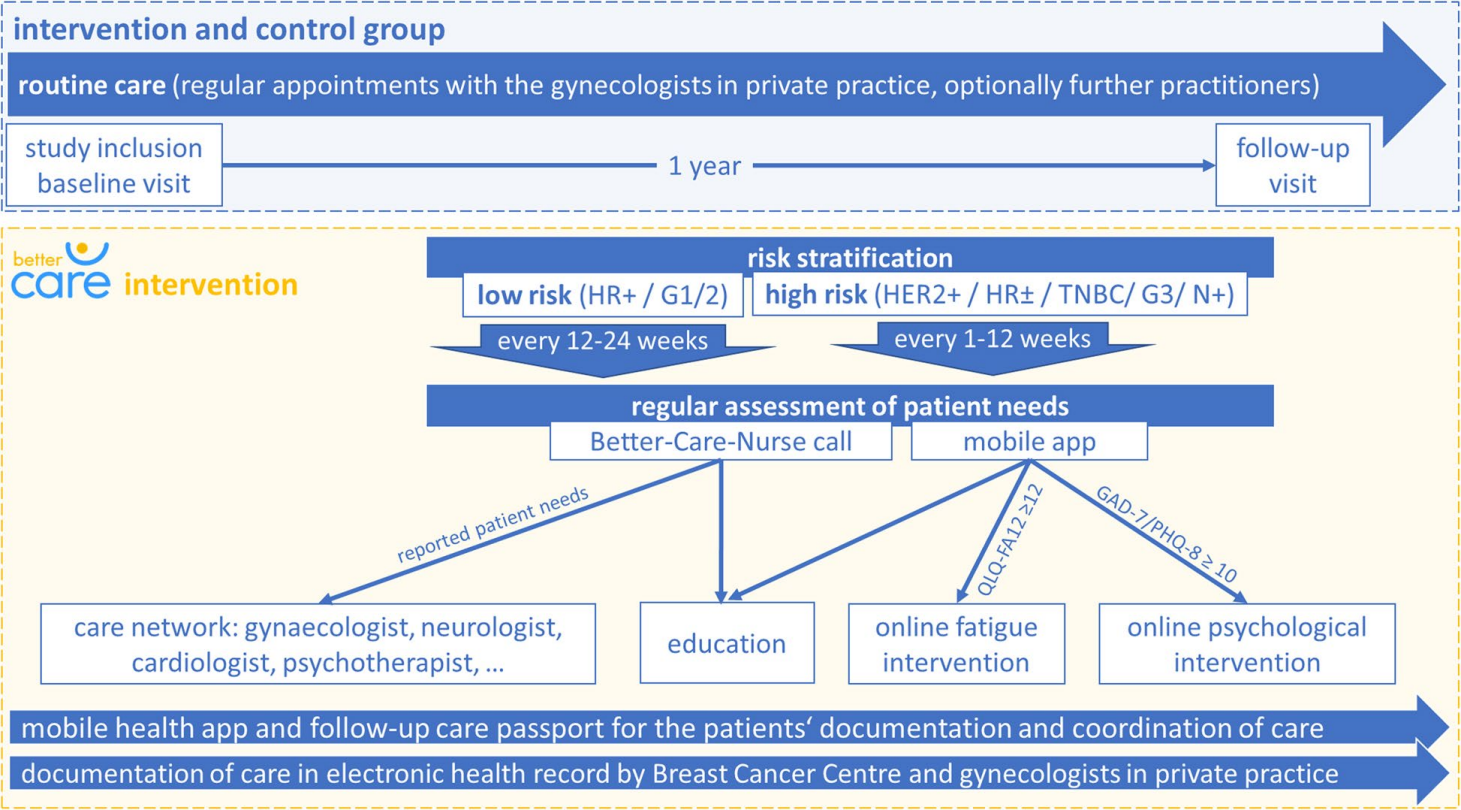
Trial course of control and intervention group in the BETTER-CARE trial. QLQ-FA-12, EORTC Quality of Life Module Measuring Cancer Related Fatigue; GAD-7, Generalised Anxiety Disorder 7-Item; PHQ-8, Patient Health Questionnaire 8-Item

Patients in the control group receive usual care according to German clinical guidelines [15]. In the intervention group, a complex intervention is established. The development of the intervention was guided by the recommendations of the Medical Research Council (MRC) framework for complex interventions [29–31]. A board of experts from the disciplines of gynaecology, epidemiology, psychology, and medical informatics was assembled, which compiled a model of the relevant outcomes and different components based on current evidence and qualitative reports from practitioners. In particular, the gaps in care identified during the development of the clinical guideline concerning needs- and risk-adapted follow-up care as well as the existing lack of networking and digitalisation of health care were considered [15, 19]. The individual components of the intervention aim to detect the needs as well as to be able to react adequately to them and the individual risk of a patient (Fig. 2). This is achieved through structural measures as well as individual behavioural components. The structural intervention components are:

- Intersectoral and multidisciplinary network of local healthcare providers coordinated by the Breast Cancer Centre, and
- The electronic health record (myoncare©) used by Breast Cancer Centre and primary follow-up care provider.

The behavioural components comprise:

- The regular assessment of patient needs by the trained study staff of the Breast Cancer Centre or via smartphone app,
- Individual use of follow-up care passport, and
- Individual use of digital applications and intervention.

Subsequently, the recruitment and procedures of the complex intervention and outcome assessment were examined in a pilot phase.

#### Breast Cancer Centre activities: needs assessment, network, and electronic health record

There is insufficient evidence that supporting patients’ self-management alone can effectively improve patient-reported outcomes, health care or clinical outcomes [32]. Therefore, with BETTER-CARE, a concept was developed in which nurses and primary care physicians are involved in coordination, needs analysis and education. Nurse-led follow-up is reported to potentially support continuity of care, provision of psychosocial support and meeting patients’ information needs [18]. In BETTER-CARE, patients are accompanied by the trained nurses (BETTER-CARE-Nurse) and physicians (BETTER-CARE-Coordinator) of their Breast Cancer Centre. They receive a regular assessment of patient needs via phone call. Phone call frequency for low-risk patients is supposed to take place every 6 months, high risk every 3 months. If patients do not use the app to answer the symptom questionnaires, the phone call frequency is increased to every 3 months for low-risk and once a month for high-risk patients.

If an unfulfilled need is detected in the assessment, patients are offered treatment in the follow-up care network by the BETTER-CARE-Coordinator (e.g. referral to the neurologist for the treatment of sensorimotor neuropathy), or information about possible support (e.g. regional support groups).

Every phone call and referral to practitioners is documented in the electronic health record. Questions and categories specifically adapted to the needs and follow-up care situation of breast cancer patients were designed in advance as part of the study. In addition, data from routine care is imported into the electronic health record via the Oncobox©, so that data from acute treatment and follow-up care can be made available collectively. The patients' gynaecologists in private practice can access their patient’s records within the framework of cooperation if the patient agrees. This enables an intersectoral exchange.

The platform of the electronic health record complies with EN ISO 13485, ISO 27001, MDR IIa, FDA and BSI standards and can be accessed online by authorised healthcare professionals. Contracts for data processing in accordance with the European General Data Protection Regulation have been concluded for both the electronic health record and the apps for patients and the data is hosted on servers in the EU.

#### Tools for the patient: digital interventions and follow-up passport

In the BETTER-CARE trial, two digital applications are available to support the patient. Existing self-management health apps for breast cancer survivors often include features such as survivor education, social networking, and symptom tracking, but studies on their effectiveness are rare and results inconclusive [33–36]. Phone- and Internet-based interventions for breast cancer patients compared to routine care improved patients’ quality of life, self-efficacy, depression, perceived stress, some somatic symptoms, and treatment adherence [33]. The development of the complex intervention was therefore based on the assumption that the Internet-based applications would influence quality of life, psychological distress, self-efficacy, and adherence to therapy. All patients of the intervention group are invited to use the PIA (“Patient-informiert-interaktiv-Arzt”; patient interactively informs doctor) Health app to assess their well-being [37]. It can be accessed via the native PIA Health app, which is available for iOS and Android in the Apple App or Google Play Store, with a study-specific registration code. Assessment frequency for low-risk patients is every 3 months and for high-risk weekly. Furthermore, the PIA Health app can be used for communicating with the Breast Cancer Centre, as authorised staff at the centre have access to the PIA Health Careportal via Internet browsers to manage their patients, and for an intervention on fatigue. The fatigue intervention is a 4-week programme developed in several rounds of interviews by the Institute for Women’s Health with the help of patient and expert interviews with the German Cancer Research Center Heidelberg, the National Center for Tumor Diseases, the University Women's Hospitals of Heidelberg and Tübingen and the Cancer Information Service. The basis for screening and intervention is the National Comprehensive Cancer Network guideline on fatigue [38–42]. Another intervention based on acceptance and commitment therapy (ACT) for cancer patients with high psychological distress (ACTonCancer intervention) consists of a 10-week intervention programme designed by experts from psychotherapy and psychology from the University of Ulm [43, 44]. ACTonCancer can be accessed online via an Internet browser or via the native ACTonCancer app which is available for iOS and Android in the Apple App or Google Play Store after participants have been registered [43, 44]. If elevated psychological distress is identified (7-item Generalised Anxiety Disorder or 8-item Patient Health Questionnaire (GAD-7/PHQ-8) score ≥ 10), ACTonCancer is suggested to these patients, and they can choose to participate. The transmission of patient data to the ACTonCancer team is encrypted via the SQL management system, enabled by the PIA Health app. Fatigue intervention is suggested if EORTC Quality of Life Module on Cancer Related Fatigue (EORTC QLQ-FA12) score ≥ 12. However, if both criteria are met, both interventions cannot be performed at the same time. Patients can start with one intervention and after 10 weeks, the other intervention option can be chosen. If the digital support is not sufficient, the BETTER-CARE-Coordinator is notified (alert function in the PIA Health app).

In addition, the intervention centres receive a follow-up passport, if none is routinely used at their centre, which they can hand out to the patient. Its use can promote patients' self-organisation and self-monitoring as well as communication with their treatment providers.

On the part of the network partners, the passport supports transparent communication with the patient and—in addition to the electronic health record—the exchange of information between the treatment partners.

### Outcomes

Health-related quality of life (HRQoL) measured by the total score of the Global health status/ QoL scale of the standardised questionnaire Quality of Life of Cancer Patients of the European Organisation for Research and Treatment of Cancer (EORTC QLQ-C30) was defined as primary outcome [45].

Secondary outcomes are quality of life, the identification of treatment adherence, treatment consequences (e.g., fatigue, neurotoxicity, cardiotoxicity), psychological comorbidities (e.g. anxiety, depression, cognitive impairment), follow-up examinations according to guidelines, participation in work life, rehospitalisation, progression-free survival, and patient satisfaction. In addition, follow-up costs in the first year will be investigated within a health economic evaluation.

Data is collected at baseline and 12 months after study inclusion via interviews and self-administered questionnaires; in addition, routine clinical data is used from the Breast Cancer Centre (Table 1). Study data is managed using REDCap© electronic data capture tools hosted at the University of Würzburg [46, 47]. Data monitoring will be performed by the Clinical Trial Centre of the University Hospital Würzburg. An independent data safety monitoring board will be established.

**Table 1:** Contents of baseline and follow-up examination

*Module*	*Target/domain*	*Instrument*
**General information**	Breast Cancer Centre	Questionnaire for employees
	Socio-demographic and economic factors	Self-administered questionnaire, WAI (partly)
**Health behaviour**	Lifestyle, sport, diet, health apps	Self-administered questionnaire
**Medical history**	Diagnosis^a^, therapy^a^, medication, adherence	Interview, routine data
	Comorbidites	CCI
**Health-related quality of life**	Disease-specific quality of life	EORTC QLQ-C30 and-BR23
	Generic quality of life	EQ-5D-5L
**Health problems**	Anxiety	GAD-7
	Depression	PHQ-8
	Psychological distress	PSS-10
	Resilience	BRS
	Somatic late effects	Interview
**Medical incidents and care**^**b**^	Follow-up care visits, rehospitalisation, overall survivorship, cancer recurrence or progression	Interview, hospital discharge papers, FIMA (partly)
**Satisfaction**	Satisfaction with care	Self-administered questionnaire
	Patient-doctor relationship, patient needs	Self-administered questionnaire
	Satisfaction with applications^b^^c^	uMARS-G
**Relatives**	Socio-demographic and caregiving information	Questionnaire for relative person
	Own anxiety and depression	PHQ-4
	Own needs and satisfaction with care	Questionnaire for relative person

*BRS* Brief Resilience Scale, *CCI* Charlson Comorbidity Index, *EORTC QLQ-C30/BR23* European Organisation for Research and Treatment of Cancer Quality of Life of Cancer Patients Core/Breast Module, *EQ-5D-5L* 5-level EuroQol five dimensions, *FIMA* Fragebogen zur Erhebung von Gesundheitsleistungen im Alter (Questionnaire for the survey of health services in old age), *GAD-7* Generalised Anxiety Disorder 7-Item, *PHQ-4/8* Patient Health Questionnaire 4/8-Item, *PSS-10* Perceived Stress Scale, *WAI* Work Ability Index, *uMARS-G* German Version of the User Mobile App Rating Scale

^a^Baseline only

^b^Follow-up only

^c^Intervention only

Additional information from the electronic patient record and the digital platforms is added to the electronic case record form (eCRF) via the interface at the 12-month follow-up. It includes information on contacts with the Breast Cancer Centre and follow-up care with the gynaecologists in private practice and information on app usage.

### Sample size

We assume that the complex intervention (BETTER-CARE) improves the follow-up care of breast cancer patients and, therefore, defined HRQoL as the primary outcome. HRQoL is assessed by the EORTC QLQ-C30 Global health status/QoL subscale after 12 months. A Cochrane Review evaluating home-based multidimensional survivorship programmes for breast cancer survivors reported a treatment effect of 4.38 points on the EORTC scale with an estimated standard deviation of 20 between the intervention and control group [48]. We assume the effect of the BETTER-CARE intervention to be comparable to the one reported in this review. Sample size calculation is based on a *t*-test for cluster-randomised trials (degrees of freedom based on number of clusters) with a significance level of 5% and a power of 90% and a fixed number of 15 clusters per group. No comparable data for the definition of interclass correlation coefficient (ICC) is available. Therefore, we assumed a lower ICC of 0.001 relying on our own experience with former cluster-randomised trials. Based on other studies with breast cancer survivors, we assume a dropout rate of 10% [49, 50]. Therefore, a total of 1140 patients, approximately 38 per cluster within 30 clusters, are supposed to be recruited. The sample size was conducted using the software PASS 2020.

### Statistical methods

Evaluations are conducted according to modified intention-to-treat, including all patients with information on the primary outcome at 12 months and who did not withdraw consent before the outcome assessment at 12 months. Descriptive analyses are done separately for the intervention and control groups using parametric or non-parametric tests according to data distribution. The primary hypothesis is tested via an univariate linear mixed effects model using Wald 95%- confidence intervals (CI) for the beta coefficient. Group is defined as a fixed factor and effects of centres as a random factor to adjust for intra-cluster-correlations. Multivariable linear mixed effects models with Wald 95%-CI of coefficients are applied to adjust for pre-defined confounders (age, risk of recurrence) in a secondary analysis of the primary outcome. Subgroup analyses such as the size of the breast cancer centre are planned. A sensitivity analysis is planned for evaluating a potential bias of centre dropout (excluding the replaced centres). Secondary outcomes are analysed in exploratory with a significance level of 5% without adjustment. In a sensitivity analysis of the primary outcome, missing values of the predictors (when missingness is higher than 5%) will be imputed using multiple imputation. Further, a descriptive comparison of baseline characteristics between dropouts and non-dropouts will be conducted. The concept of evaluating economic effects and costs is based on an incremental analysis comparing intervention to control. The analytical approaches will take the form of cost-effectiveness and cost-utility analysis. Based on trial evidence, additional costs of the complex intervention compared to routine care are put in relation to the gained quality-adjusted life years (or effect measure). In the primary economic analysis, costs will be valued from the societal perspective. In supplementary analyses, costs will be valued (1) from a combined perspective of the statutory health insurance (“Gesetzliche Krankenversicherung”) and the statutory nursing insurance (“Pflegeversicherung”) and (2) from the perspective of the statutory health insurance. Evaluations are conducted according to intention-to-treat. Statistics software SAS and R are used for evaluating the pilot phase, primary and secondary outcomes. The evaluation of health economics is conducted using Stata 17.

### Process evaluation

During the study, a process evaluation is carried out in accordance with the principles of the MRC to examine the implementation of the study intervention [51]. For this purpose, a random selection of approximately 15 patients from the intervention centres, stratified by centre, will be interviewed 3–6 months after study inclusion. In addition, approximately one healthcare professional from each intervention centre who is involved in the intervention will be interviewed. The interviews will cover the following topics: Telephone symptom and needs assessment, referral to the care network, documentation and information exchange between gynaecologists and centres in electronic health records, and apps. In addition, the screening logs with reasons for non-inclusion of certain patient groups and an online survey of the participating intervention centres on the extent of the implementation of the intervention will be evaluated.

### Pilot phase

The aim of the pilot phase was to record the average duration of the baseline survey and the implementation of the different intervention components. The pilot feasibility trial was developed according to the CONSORT 2010 statement extension to randomised pilot and feasibility trials [52]. A descriptive overview of the population and the recruitment rate will be given. In addition, the feasibility and acceptability of the digital applications were assessed, and the data flows were to be tested.

One of the control centres and one of the intervention centres were supposed to recruit up to 20 patients for the pilot phase. The patients then could not be enrolled in the main study. They received the baseline visit of the main study and answered questions on the acceptance and comprehensibility of the study information and questionnaires. The patients in the intervention group went through a test phase of the intervention components for about 4 weeks. They then rated the personal relevance, comprehensibility, and usability of the intervention components. The apps were rated using the German version of the Mobile Application Rating Scale for users (uMARS-G) [53]. A cooperation with the gynaecologists in private practice, like it is planned in the main study, was not initiated. In the pilot phase, the gynaecologists received the information sheet and a questionnaire on the acceptance of the study concept and the comprehensibility of the information. In addition, one relative person of each patient had the opportunity to fill out the relatives’ questionnaire of the main study and to evaluate the comprehensibility and feasibility. Finally, the study staff answered questions about the procedure and the time required.

## Results

### Recruitment, randomisation and training of centres

**Fig. 3:**
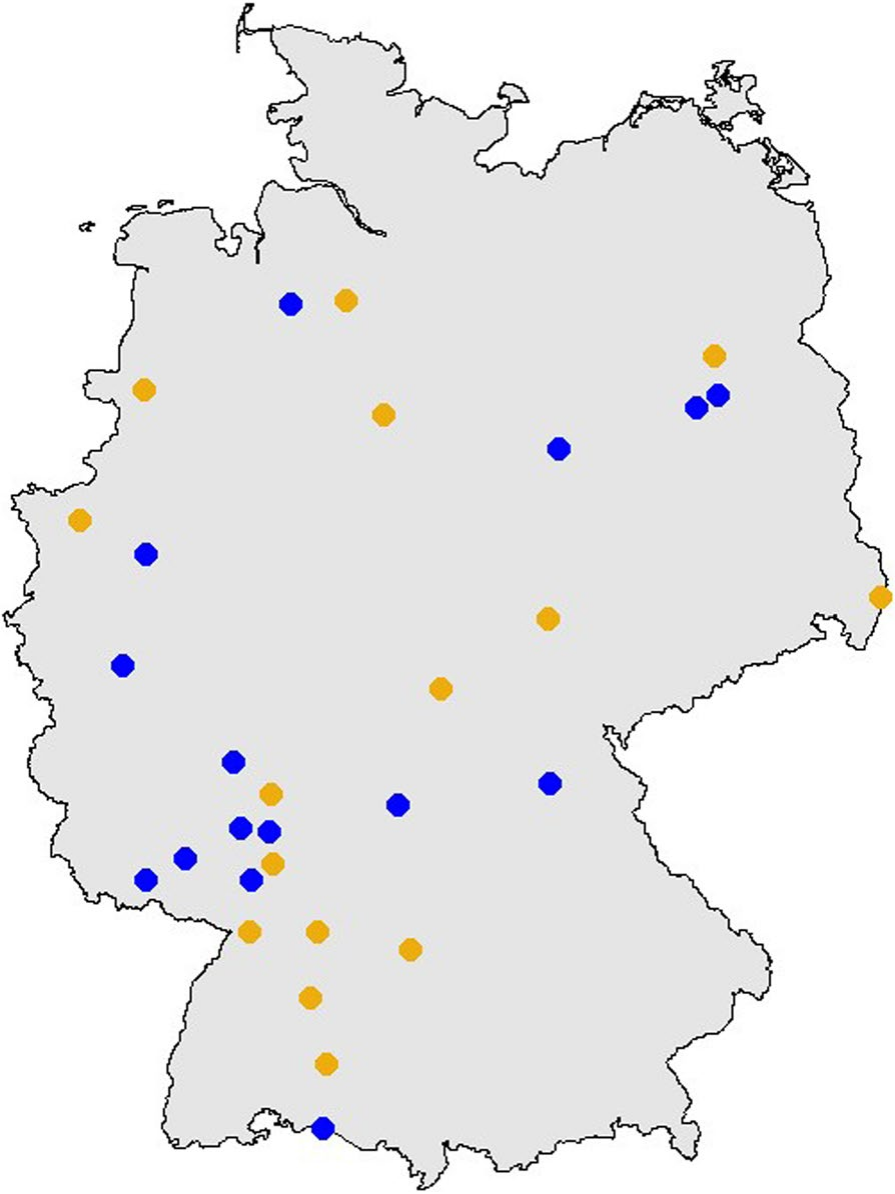
The 30 participating centres of the BETTER-CARE trial: 15 intervention and 15 control regions. The graphic was created with R version 4.2.2 ©2022 The R Foundation for Statistical Computing, packages maps and mapadata

We invited 181 Breast Cancer Centres, 64 communicated their interest in participating at the study and 30 centres were successfully recruited (Fig. 3). Randomisation into 15 intervention and 15 control centres was finalised in March 2022 using SAS® software (Version 9.4). Two centres from the intervention and one centre from the control group dropped out after randomisation, but before the start of recruitment, and were replaced by a substitute centre. The reason for drop-out in all three centres was the short-term shortage of study personnel.

The study was approved in April 2022 by the central ethics committee in Würzburg (registry number 12/22-sc). All centres obtained approval from the local ethics committees before study recruitment.

Recruitment of patients into the main study started after all centres were successfully enrolled in March 2023. All centres received a 1-day training (online or in person) for recruitment and use of eCRF and questionnaires. The intervention centres received at least one additional day of face-to-face training on the technique of motivational interviewing and two online training sessions on the digital components of the intervention. The Breast Cancer Centres received the study documents in an investigator site file and additional materials online via cloud. Information about the BETTER-CARE trial for patients and potentially cooperating physicians was made available on the project’s website www.better-care.health.

### Pilot feasibility trial

Sixteen patients were recruited during the pilot phase from December 2022 to February 2023, seven in the intervention and nine in the control centre. In the intervention group, one baseline questionnaire and two follow-up questionnaires were missing from patients, one relative answered the questionnaire and two gynaecologists. In the control group, questionnaires were available for all patients, but no relative answered a questionnaire.

All participants of the pilot study were female patients with a breast cancer diagnosis 1 to 25 months before inclusion (Table 2). Fifteen patients answered questions on their nationality and living situation, of whom two were not of German nationality, one was eligible to receive health care assistance, eleven had their own children and lived with a partner or family, two lived alone, one person was in hospital and one in a shared apartment.

**Table 2:** Descriptive scores from the questionnaires of the pilot phase

*Outcome*	*Total*	*Median*	*Range*
***General characteristics***			
Age (years)	16	44.0	29.0–73.0
Time since diagnosis (weeks)	16	39.9	4.6–98.0
Body-mass-index	12	26.8	13.2–30.8
***Pilot phase***			
Duration of answering baseline questionnaire (minutes)	15	51.0	17.0–268.0
Overall evaluation of the baseline visit (1 = very good, 6 = insufficient)	14	2.0	1.0–3.0

The participant information was handed out during the primary treatment of the patients at the centre and a separate appointment was made for consent and interview if interested. The information materials comprised 6 pages in the control group and 8 pages in the intervention group, as well as a flyer with a brief description and a link to the project website. The study staff then needed 5–15 min for the information session, answering questions and obtaining informed consent. No problems were reported in recruiting patients for the control group, even though they had no direct benefit from participating in the study. The duration of the baseline interview was 5 to 60 min. The study staff rated the comprehensibility of the questionnaires generally as good. The patients rated the comprehensibility and length of the information material and the comprehensibility and relevance of questions as very good to moderate.

Fifteen patients were given the survey for a related person, but only one person completed the survey. The relative was between 35 and 49 years old and supported the breast cancer patient on average 20 h per week. The personal relevance of the questionnaire was rated as moderate, as the relative did not feel the need of support but was asked questions about this (e.g. medical care, social and emotional support, socio-legal advice). Overall, the study information and the questionnaire were rated as very good.

The upload of routine data from Oncobox© to the electronic patient record was successfully tested. The import into the eCRF could not yet be tested.

In the intervention group, patients were asked to answer an online survey on the intervention components 4 to 6 weeks after baseline assessment, which five of the seven patients did. In general, the applicability and relevance of the intervention was rated as very good to moderate by the study staff and patients. The personal relevance and support of the needs assessment via phone call, which every intervention patient of the pilot phase received once, was rated as very good to moderate. For one participant, the phone call prompted further treatment.

Regarding the applications, one person needed assistance with the installation and therefore contacted the Breast Cancer Centre. Three participants used the PIA Health app at least five times, one participant less frequently and one not at all. Reasons for the low usage were, for one patient, that the need for support could be covered quickly by the app, and, for the other patient, it was not possible to use it with her own device. On the uMARS-G overall score (from 1 = bad to 5 = good), the PIA health app was rated with a median of 4.3 (range = 3.7–4.4). The fatigue intervention, which could be accessed via the PIA Health app when reporting a higher fatigue score, was tested separately from the other functions of the PIA Health app. The intervention was piloted internally by psychologists, physicians, and project staff (*n* = 6, age between 27 and 54 years) and was mainly evaluated in terms of functionality, usability and relevance of content. Individual changes to the text and improvements to the functionality were then implemented. The ACTonCancer app was not used by any of the participants of the BETTER-CARE pilot study, as no need was identified by the questionnaire responses. Therefore, the app was also tested by a separately recruited patient who rated the app with an overall uMARS-G score of 3.9. The exchange of information between the two intervention platforms to refer patients from the PIA Health app to the ACTonCancer intervention has been successfully implemented.

Six gynaecological practices received the survey on cooperation in the intervention, but only two gynaecologists answered. No telephone call was made to the Breast Cancer Centre, but the documents received were rated as difficult to understand. The relevance of the intervention was rated as medium. Both gynaecologists stated that they were unable to establish cooperation in the intervention due to a lack of capacity. They would not use the electronic health record because they also lacked capacity or did not benefit from it. However, one of the practices would forward documents on follow-up care to the centre to be uploaded into the electronic health record.

## Discussion

The BETTER-CARE intervention complements the existing specialist-led routine follow-up breast cancer care in Germany with a primary care-coordinated aftercare network, nurse-led assessment of needs via a regular interview, symptom monitoring via app and digital support services for fatigue and elevated psychological distress. Currently, a variety of cancer follow-up care models are being investigated, including approaches that are used as an adjunct to routine follow-up and stand-alone follow-up care, that is led by specialists, primary care providers, or nurses [16, 17]. Additionally, mixed models, home-based programmes, and patient-initiated approaches exist [16, 17, 32, 48, 54]. There is already good evidence for the effectiveness of individual interventions in meeting patients’ needs in breast cancer follow-up [8]. The feasibility of these concepts depends heavily on the local healthcare system, financial constraints, patient population, and practitioners [16, 17]. Therefore, concepts need to be implemented that clearly define how and by whom patients' needs can be identified and how care is coordinated.

### Feasibility

Feasibility of the BETTER-CARE trial was tested in a 2-month pilot phase. The following experiences from the pilot phase were incorporated into the training of the study centres: Approaching patients during primary treatment because the patient is on site at the centre at this time and actively arranging an appointment for study inclusion after the end of primary treatment. In addition, the study staff were advised to schedule sufficient time for the baseline visit and a control mechanism was set up to check the completeness of the patients' online surveys. Some questions in the interview were reworded to make them easier to understand, but the content of the baseline visit was not adapted following the positive feedback. Even though only one relative person answered the questionnaire, we decided against implementing a more complex procedure with reminders and monitoring of the relatives’ survey, as the relatives’ situation was not the focus of the study. There was no referral between the PIA Health app and the two interventions during the pilot phase. The used cut-offs were discussed but not adjusted based on the existing literature. The technical aspects of forwarding to the ACTonCancer intervention were checked and corrected at the beginning of the main study. The documents for the gynaecological practices in the intervention group were reformulated to ensure better comprehensibility and to increase motivation for cooperation, and reviewed by one gynaecologist. After exchanging ideas with the pilot centre, the procedure for establishing cooperation was adapted so that direct contact takes place between the centre and the gynaecological practice if there is no response to the written invitation after a few weeks. The development of the follow-up care network in the intervention regions was not part of the pilot phase and will be evaluated in the main study and process evaluation. Data import into the eCRF will be tested separately and will have to be closely monitored. Recruitment for the main study successfully started in March 2023 and will be completed in June 2024. The completion of follow-up is planned for June 2025.

### Ethics and dissemination

In the intervention group, patients receive support services from their centre on treatment options and in finding suitable therapists, as well as through a follow-up passport and two applications. The study centre must notify the central study management of any study discontinuation or reported side effects. Usual care will continue for all participants, so there are no additional risks. However, the visits and the intervention are associated with additional effort. Additionally, all participants will travel to the centre to carry out the on-site visits. Therefore, travel accident insurance is provided, and participants can receive a travel allowance. All study-relevant data are stored in a central database and are available to the consortium for subsequent evaluation. For this purpose, a cooperation and data protection agreement was concluded between the consortium partners.

### Strengths and limitations

Strengths of the BETTER-CARE trial are the use of cluster randomisation, as the intervention includes structural elements of the study centres. Therefore, 30 centres from all over Germany were included. The complex intervention enables the inclusion of various relevant aspects of follow-up care. The basis of the intervention is the development of a model in which the mechanisms of action and the context are strongly considered.

One limitation of the pilot phase is the small number of patients and short follow-up duration. Only a limited number of patients could be included, as the patients in the pilot phase could not participate in the main study, but the centres subsequently had to recruit patients for the main study. The number of pilot patients in the intervention group was therefore not sufficient to test the referral from the PIA Health app to the ACTonCancer intervention, as no patient exceeded the predefined cut-off. We also received only a small number of responses from relatives and gynaecologists in private practice, which made it difficult to draw conclusions about these groups as a whole. Nevertheless, constructive feedback was obtained, which was used to optimise the recruitment process, endpoint recording, symptom and needs assessment via telephone and the establishment of cooperation with gynaecologists. Furthermore, the information gained from the pilot phase was passed on in the training courses. For similar complex interventions, however, we recommend a longer pilot and feasibility phase of the intervention and more time for implementing changes and training. A limitation of the study concept is that three centres assigned to the intervention or control group dropped out after their randomisation. Although it was possible to replace these centres, a self-selection bias cannot be ruled out for the centres that dropped out, which is why a sensitivity analysis is planned in the evaluation of the main study. The requirement for the centre to be able to provide sufficient staff for study implementation may have led to a selection bias of higher performing centres. Moreover, the components of the complex intervention can only be evaluated as a whole construct. This can make implementation in routine care more difficult.

## Conclusions

In summary, current research on cancer follow-up care highlights the need of developing an evidence base for survivorship strategies and studying their implementation, both in terms of technical training and financing [16]. By implementing a complex intervention, BETTER-CARE is investigating individualised needs- and risk-adapted breast cancer follow-up care in Germany. Successful outputs could be implemented in German routine follow-up care.

## Supplementary Information

Supplementary Material 1.

## Data Availability

The individual participant data, after deindentification, that support the findings of the pilot trial, study protocol and informed consent materials can be made available following publication from the corresponding author in its original language to qualified investigators upon reasonable request.

## Abbreviations

ACT: Acceptance and commitment therapy
BETTER-CARE: BrEasT cancer afTER CARE follow up and programme
eCRF: Electronic case record form
EORTC QLQ-C30: European Organisation for Research and Treatment of Cancer Quality of Life of Cancer Patients questionnaire
EORTC QLQ-FA12: EORTC Quality of Life Module on Cancer Related Fatigue
GAD-7: 7-Item Generalised Anxiety Disorder Questionnaire
HRQoL: Health-related quality of life
ICC: Interclass Correlation Coefficient
MRC: Medical Research Council
PHQ-8: 8-Item Patient Health Questionnaire
PIA: Patient interactively informs doctor
uMARS: Mobile Application Rating Scale for users
