# Correction: The BrEasT cancer afTER-CARE (BETTER-CARE) programme to improve breast cancer follow-up: design and feasibility study results of a cluster-randomised complex intervention trial

**DOI:** 10.1186/s13063-026-09858-2

**Published:** 2026-06-30

**Authors:** Anna Schäfer, Julia Wendel, Isabella Franke, Armin Bauer, Harald Baumeister, Eileen Bendig, Sara Y. Brucker, Thomas M. Deutsch, Patricia Garatva, Kirsten Haas, Lorenz Heil, Klemens Hügen, Helena Manger, Rüdiger Pryss, Viktoria Rücker, Jessica Salmen, Andrea Szczesny, Carsten Vogel, Markus Wallwiener, Achim Wöckel, Peter U. Heuschmann, the BETTER-CARE Study Group

**Affiliations:** 1 Institute of Clinical Epidemiology and Biometry, Julius-Maximilians-Universität Würzburg, Würzburg, Germany; 2 Department of Gynecology and Obstetrics, University Hospital Würzburg, Würzburg, Germany; 3 Institute Women’s Health GmbH, Tübingen, Germany; 4 Department of Clinical Psychology and Psychotherapy, Institute of Psychology and Education, University of Ulm, Ulm, Germany; 5 Department of Women’s Health, University Women’s Hospital Tübingen, Tübingen, Germany; 6 University Hospital Heidelberg, Heidelberg, Germany; 7 University Hospital Würzburg, Clinical Trial Center Würzburg, Würzburg, Germany; 8 Faculty of Business Management and Economics, Julius-Maximilians-Universität Würzburg, Würzburg, Germany; 9 Institute for Medical Data Science, University Hospital Würzburg, Würzburg, Germany; 10 University Women’s Hospital Halle, Halle, Germany

**Correction: Trials 25, 767 (2024)**

**https://doi.org/10.1186/s13063-024-08614-8**

Following the publication of the original article [1], we were notified that the first author’s last name needs to be changed from Horn to Schäfer.

The original article has been corrected.
