# Supplementary material for: The BrEasT cancer afTER-CARE (BETTER-CARE) programme to improve breast cancer follow-up: design and feasibility study results of a cluster-randomised complex intervention trial
Source: Trials. 2024 Nov 14;25:767. doi: 10.1186/s13063-024-08614-8 (PMC11566082; doi:10.1186/s13063-024-08614-8)
Supplement: Supplementary file 1 — Supplementary Material 1. [file 13063_2024_8614_MOESM1_ESM.doc]

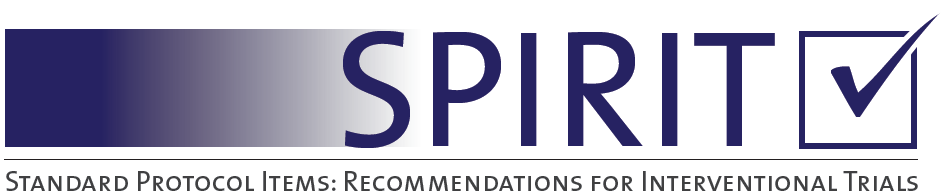


SPIRIT 2013 Checklist for the BETTER-CARE trial: Recommended items to address in a clinical trial protocol and related documents*

| Section/item | ItemNo | Description |
| --- | --- | --- |
| **Administrative information** | | |
| Title | 1 | Improving breast cancer follow-up care with BETTER-CARE (BrEasT cancer afTER CARE follow up and programme): study protocol and pilot feasibility study of a cluster-randomised intervention trial |
| Trial registration | 2a | DRKS00028840 |
|  | 2b | available from: <https://drks.de/search/de/trial/DRKS00028840> |
| Protocol version | 3 | V1.6 from December 01, 2023 |
| Funding | 4 | The German Innovation Fund of the the Federal Joint Committee (“Innovationsfonds des Gemeinsamen Bundesausschusses”, grant number 01NVF20015). |
| Roles and responsibilities | 5a | Names, affiliations, and roles of protocol contributors: Anna Schäfer (coordination)^1^, Julia Wendel (coordination)^1^, Isabella Franke (coordination)^2^, Armin Bauer (PI)³, Harald Baumeister (PI)⁴, Eileen Bendig (coordination sub-project)⁴, Sara Brucker (PI)⁵, Thomas Deutsch (coordination sub-project)⁶, Patricia Garatva (coordination sub-project)⁴, Kirsten Haas (coordination)^1^, Lorenz Heil (coordination sub-project)⁸, Klemens Hügen (PI)^7^, Helena Manger (coordination sub-project)⁸, Rüdiger Pryss (PI)^1,9^, Vikotria Rücker (biometry)^1^, Jessica Salmen (coordination)^2^, Andrea Szczesny (PI)⁸, Carsten Vogel (coordination sub-project)^1,9^, Markus Wallwiener (PI)⁶, Achim Wöckel (PI, lead member of consortium)^2^, Peter U Heuschmann (PI)^1,7,9^  ¹ Julius-Maximilians-Universität Würzburg, Institute of Clinical Epidemiology and Biometry, Würzburg, Germany  ² University Hospital Würzburg, Department of Gynecology and Obstetrics, Würzburg, Germany  ³ Institute Women’s Health GmbH, Tübingen, Germany  ⁴ University of Ulm, Department of Clinical Psychology and Psychotherapy, Institute of Psychology and Education, Ulm, Germany  ⁵ University Hospital Tübingen, Tübingen, Germany  ⁶ University Hospital Heidelberg, Heidelberg, Germany  ⁷ University Hospital Würzburg, Clinical Trial Center Würzburg, Würzburg, Germany  ⁸ Julius-Maximilians-Universität Würzburg, Faculty of Business Management and Economics, Würzburg, Germany  ^9^ University Hospital Würzburg, Institute for medical Data Science, Würzburg, Germany |
|  | 5b | Name and contact information for the trial sponsor: Achim Wöckel, lead member of consortium, University Hospital Würzburg, Department of Gynecology and Obstetrics, Würzburg, Germany  [better-care@ukw.de](mailto:better-care@ukw.de) |
|  | 5c | Role of study sponsor: study design; collection, management, analysis, and interpretation of data; writing of the report, decision to submit the report for publication. Role of funders: Directive to submit the report for publication, but no decision on how and where. |
|  | 5d | Composition, roles, and responsibilities of:  coordinating centre (University Hospital Würzburg, Department of Gynecology and Obstetrics): recruitment of participating centres, development of intervention, coordination;  consortium: study planning and coordination of the subprojects, evaluation and publication;  steering committee, endpoint adjudication committee: not applicable;  data management (Julius-Maximilians-Universität Würzburg, Institute of Clinical Epidemiology and Biometry): coordination of ethical approvement, study protocol, training, coordination. |
| Introduction |  |  |
| Background and rationale | 6a | Within the framework of the German guideline of diagnosis and care of breast cancer (“AWMF S3 Leitlinie”) a needs- and risk-adapted follow-up is recommended after completion of primary treatment. Up to now, Germany has lacked an individualised breast cancer follow-up concept. In the BETTER-CARE study, a multidisciplinary care network is to be developed, and supplemented by supporting digital applications, to provide needs- and risk-based individualised breast cancer follow-up care. The complex intervention for patients after primary breast cancer is supposed to improve health-related quality of life |
|  | 6b | As in the control condition, patients in the intervention group are supposed to receive standard care according to German guidelines. |
| Objectives | 7 | Primary objective of the study:  - To investigate the increase in health related quality of life (HRQoL) due to the treatment of patients in the new BETTER-CARE form of care compared to usual care.  Secondary objectives of the study:  - To investigate risk-specific medium- and long-term effects of the intervention. For example, a higher adherence to therapy can be expected in low-risk patients due to educational elements. In the group of high-risk patients, a reduction of therapy-related side effects (e.g. fatigue, cardiotoxicity) as well as an earlier intervention for the therapy of psychological comorbidities can be expected.  - Investigation of the effects of the intervention on participation in working and social life (re-entry into working life, lower rates of incapacity to work and reduced restrictions in activities of daily living), re-hospitalisations and follow-up costs. |
| Trial design | 8 | BETTER-CARE is a parallel-arm cluster-randomised superiority controlled trial, allocation ratio is 1:1. |
| Methods: Participants, interventions, and outcomes | | |
| Study setting | 9 | certified German Breast Units that could theoretically export routine data via the Onkozert© software (see [www.better-care.health](http://www.better-care.health/)) |
| Eligibility criteria | 10 | Inclusion criteria are defined as 18 years and older and not being restricted to specific gender. Further criteria are the end of curative primary treatment and consent to participation. Exclusion criteria comprise the diagnosis of metastatic breast carcinoma (palliative therapy approach) and lack of digital infrastructure. Patients are additionally excluded when participating in another structured follow-up programme with an intervention via digital platforms. |
| Interventions | 11a | The complex BETTER-CARE intervention can be divided into structural and behavioural intervention components.  The structural intervention components are:  • intersectoral and multidisciplinary networking of local healthcare providers coordinated by the Breast Unit, and  • the electronic patient record file (myoncare©) used by Breast Unit and primary follow-up care provider.  The behavioural components comprise:  • the regular assessment of patient needs by the trained study staff of the Breast Unit or via smartphone app,  • individual use of follow-up care passport, and  • individual use of digital applications PIA health app or ACTonCancer intervention |
|  | 11b | Criteria for discontinuing or modifying allocated interventions for a given trial participant: participant request. |
|  | 11c | The better-care-nurses were trained to contact the patients regularly and to motivate them. Adherence to the behavioural intervention components is documented in the digital platforms. Adherence of the study centre to the structural intervention components is checked as part of the process evaluation. |
|  | 11d | Interventions that are prohibited during the trial: structured follow-up programme with an intervention via digital platforms (analogous to electronic health record, PIA heath application and platform, or ACTonCancer intervention of the BETTER-CARE program). |
| Outcomes | 12 | Primary outcome: health-related quality of life (EORTC QLQ-C30 global health subscale).  Secondary outcomes: health-related quality of life (EORTC QLQ-C30 other subscales, EQ-5D-5L), participation in social and working life, therapy-related toxicities, psychological distress, adherence to guidelines, compliance with appropriate adjuvant therapies rehospitalization, progression free survival, patient satisfaction, follow-up costs. |
| Participant timeline | 13 | Baseline-visit directly after study inclusion and follow-up at 12 months after study inclusion. |
| Sample size | 14 | A Cochrane Review evaluating home-based multidimensional survivorship programmes reports a treatment effect of 4.38 points on the EORTC scale with an estimated standard deviation of 20 between intervention and control group. We assume the effect of the BETTER-CARE intervention to be comparable to the one reported in the review. Sample size calculation is based on a t-test for cluster-randomised trials with a significance level of 5% and a power of 90%. No comparable data for the definition of Interclass Correlation Coefficient (ICC) is available. We assume an ICC of 0.001 relying on our own experience with former cluster-randomised trials. Based on other studies with breast cancer survivors, we assume a drop-out rate of 10%. Therefore, a total of 1,140 patients, approximately 38 per cluster, are supposed to be recruited. |
| Recruitment | 15 | Strategies for achieving adequate participant enrolment to reach target sample size are the training of the study centres, press releases, and payment of a travel allowance. |
| **Methods: Assignment of interventions (for controlled trials)** | | |
| Allocation: |  |  |
| Sequence generation | 16a | The participating Breast Units were randomly assigned to intervention or control group using block randomization with variable block sizes of 4 and 6 in SAS Version 9.4. |
| Allocation concealment mechanism | 16b | The allocation was carried out by a staff member who was not involved in the recruitment and training of the centres, and the allocation remained under lock in a restricted folder on a drive until a group of centres had signed the contract and were informed of their group. The centres were instructed not to disclose their allocation to anyone until all allocations were fully disclosed. |
| Implementation | 16c | As this is a cluster randomised trial, no allocation sequence for participants is applied. Depending on the allocation of the centre, all patients recruited by the study team will be in the interventional or control group. |
| Blinding (masking) | 17a | Blinding of trial participants and care providers is not possible due to the intervention characteristics of the cluster randomized trial. Blinding of outcome assessors and of data analysts is not applicable. |
|  | 17b | Circumstances under which unblinding is permissible: Not applicable. |
| **Methods: Data collection, management, and analysis** | | |
| Data collection methods | 18a | Data is collected in the electronic case record form (eCRF) using REDCap© at zero and twelve months after study inclusion via interview and self-administered, standardized questionnaires and via interface from the routine documentation at the Breast Unit. |
|  | 18b | Outcome data to be collected for participants who discontinue the study participation: progression free survival. |
| Data management | 19 | Data entry in eCRF using REDCap© or import via interface from the routine documentation at the Breast Unit, processes to promote data quality (monitoring visit with source data verification; range checks for data values). |
| Statistical methods | 20a | Descriptive analyses are done separately for intervention and control group using parametric or non-parametric tests according to data distribution. The primary hypothesis is tested via linear univariate mixed effects model. Intervention and control group are defined as fixed factor and effects of centres as random factor to adjust for intra-cluster-correlations. The concept of evaluating economic effects and costs is based on an incremental analysis comparing intervention to control. Additional costs of the complex intervention compared to routine care are put in relation to the gained Quality-Adjusted Life Years. Statistic software SAS and R are used for evaluating primary and secondary outcomes. The evaluation of health economics is conducted using Stata 17. |
|  | 20b | Secondary outcomes are analysed exploratory with a significance level of 5% without adjustment. |
|  | 20c | Evaluations are conducted according to intention-to-treat principle on the full analysis set. Multiple imputation to handle missing data will be conducted as a sensitivity analysis for primary and secondary outcomes. |
| **Methods: Monitoring** | | |
| Data monitoring | 21a | A data safety monitoring board is to be set up. Members are independent from the sponsor and competing interests. Regular meetings with report take place. |
|  | 21b | No interim analyses are planned or stopping guidelines defined. |
| Harms | 22 | Serious adverse events must be reported by the local principal investigator to the central principal investigator. The data safety monitoring board checks these. |
| Auditing | 23 | The central study coordination checks the recruitment by the centres on a monthly basis. With regard to the intervention, a process evaluation takes place; an audit does not take place. |
| Ethics and dissemination | | |
| Research ethics approval | 24 | The study was approved in April 2022 by the central ethics committee in Würzburg (registry number 12/22-sc). All recruiting centres will obtain approval by the local ethics committees before study initiation. |
| Protocol amendments | 25 | Important protocol modifications (eg, changes to eligibility criteria, outcomes, analyses) are communicated to the ethics committee as an amendment to the review, to the consortium and project partners as well as study centres via newsletter, to registers and journals via the digital channels specified there. |
| Consent or assent | 26a | Inclusion and exclusion criteria are to be checked by the local medical invesitgator, trained study staff can obtain informed consent. |
|  | 26b | Additional consent provisions for collection and use of participant data and biological specimens in ancillary studies: not applicable. |
| Confidentiality | 27 | The research data from the baseline and follow-up visits required for the study are entered directly into the REDCap(C) database by the centres; the data from the electronic patient record (ePA) and the digital applications for the patients are imported into REDCap(C) pseudonymously and stored at the Institute of Epidemiology and Biometry at the University of Würzburg (IKE-B). Access is restricted to a defined group of persons with an individual definition of read and write rights. Pseudonyms can only be assigned to real names at the study centres. For evaluation purposes, the pseudonym is replaced by a consecutive number. |
| Declaration of interests | 28 | The authors declared the following potential conﬂicts of interest with respect to the research, authorship, and/or publication of this article: ASc, JW, IF, AB, TMD, PG, KiH, LH, KH, HM, VR, AS, CV declare that they or a family member have not had any economic or personal ties in the last 3 years. HB reports research grants from the Federal Joint Committee (G-BA) within the Innovationfond, during the conduct of the study; royalties or licenses from HelloBetter, consulting fees from Roche, speaker honoraria as member of the digital agenda BPtK and for E-Mental-Health Lectures DRV-BW, SAMA, Medical Centre Göppingen, and Participation on a Data Safety Monitoring Board for IMMERSE EU-Project. EB reports Honoraria for a workshop at “6. Hamburger Tag der Psychoonkologie ´Krebs und Innovation´“. SYB reports research grants from the Federal Joint Committee (G-BA) within the Innovationfond, during the conduct of the study; Advisory board honoraria and speaker fees received from: Medtronic, Sanofi, Köhler, AstraZeneca, Lilly, MSD, Hologic, Roche. JeS reports speaker honoraria received from AstraZeneca, Clovis Oncology, Dajichi Sanko, GILEDA, GSK, Lilly, Novartis Pharma GmbH, Pfizer, SEAGEN; support for attending meetings and/or travel received from Dajichi Sanko, GILEDA, Lilly. MW reports research grants from the Federal Joint Committee (G-BA) within the Innovationfond, during the conduct of the study; personal fees, non-ﬁnancial support, speaker fees, consultancy honoraria or grants received from Novartis, Pfizer, Roche, Celgene, Daiichi Sankyo, AstraZeneca. RP reports research grants from the Federal Joint Committee (G-BA) within the Innovationfond, during the conduct of the study. AW reports research grants from the Federal Joint Committee (G-BA) within the Innovationfond, during the conduct of the study; consulting fees received from AstraZeneca, Celgene, Eisai, Lilly, Novartis, Pfizer, Roche, MSD, Pierre Fabre, Clovis, Organon, Seagen, Exact Sciences, Gilead, Dajichi Sanko; speaker honoraria received from Aurikamed and Onkowissen; support for attending meetings and/or travel received from Pfizer, Dajichi Sanko, Seagan; leadership or fiduciary role in AGO, S3-Guideline Breast Cancer, German Society of Breast Cancer, BGGF. PUH reports research grants from the Federal Joint Committee (G-BA) within the Innovationfond, during the conduct of the study; research grants from the German Ministry of Research and Education, Federal Joint Committee (G-BA), European Union, German Parkinson Society, University Hospital Würzburg, German Heart Foundation, German Research Foundation, Bavarian State, German Cancer Aid, Charité – Universitätsmedizin Berlin (within Mondafis; supported by an unrestricted research grant to the Charité from Bayer), University Göttingen (within FIND-AF randomized; supported by an unrestricted research grant to the University Göttingen from Boehringer-Ingelheim), University Hospital Heidelberg (within RASUNOA-prime; supported by an unrestricted research grant to the University Hospital Heidelberg from Bayer, BMS, Boehringer-Ingelheim, Daiichi Sankyo), outside the submitted work. |
| Access to data | 29 | Access to the dataset will be made available to qualified investigators upon reasonable request. |
| Ancillary and post-trial care | 30 | Not applicable. |
| Dissemination policy | 31a | Plans for investigators and sponsor to communicate trial results to participants, healthcare professionals, the public, and other relevant groups: scientific and press publication. |
|  | 31b | A study group of the participating consortium partners, cooperation partners and centres will be established. |
|  | 31c | Access to the full protocol, participant-level dataset, and statistical code will be made available to qualified investigators upon reasonable request. |
| Appendices |  |  |
| Informed consent materials | 32 | Informed consent materials can be made available in its original language to qualified investigators upon reasonable request. |
| Biological specimens | 33 | not applicable |

*It is strongly recommended that this checklist be read in conjunction with the SPIRIT 2013 Explanation & Elaboration for important clarification on the items. Amendments to the protocol should be tracked and dated. The SPIRIT checklist is copyrighted by the SPIRIT Group under the Creative Commons “[Attribution-NonCommercial-NoDerivs 3.0 Unported](http://www.creativecommons.org/licenses/by-nc-nd/3.0/)” license.
